# Delirium Screening in Aphasic Patients With the Intensive Care Delirium Screening Checklist (ICDSC): A Prospective Cohort Study

**DOI:** 10.3389/fneur.2019.01198

**Published:** 2019-11-12

**Authors:** Christian Boßelmann, Jan Zurloh, Maria-Ioanna Stefanou, Vera Stadler, Yvonne Weber, Holger Lerche, Sven Poli, Ulf Ziemann, Annerose Mengel

**Affiliations:** ^1^Department of Neurology and Epileptology, Hertie-Institute for Clinical Brain Research, Eberhard-Karls University of Tübingen, Tübingen, Germany; ^2^Department of Neurology and Stroke, Hertie-Institute for Clinical Brain Research, Eberhard-Karls University of Tübingen, Tübingen, Germany

**Keywords:** stroke, intracerebral hemorrhage, post-stroke delirium, post-stroke aphasia, intensive care delirium screening checklist

## Abstract

**Background:** Ten to thirty percent of stroke patients suffer from post-stroke delirium. This leads to a longer hospital stay and increased mortality. Therefore, early detection and treatment are needed. All established delirium screening tools require some degree of language function. We sought to investigate whether the Intensive Care Delirium Screening Checklist (ICDSC) is suitable for delirium screening in patients with post-stroke aphasia.

**Methods:** A prospective cohort study was carried out in adult patients consecutively admitted to the Stroke Unit of University Hospital Tuebingen, between July 2017 and December 2018. The index test, ICDSC, was compared with the DSM-V diagnostic criteria as reference standard. Measures of diagnostic precision and the degree of agreement were obtained.

**Results:** Three hundred and forty six patients were included in the analysis. Aphasia was present in 231 (66.8%) and absent in 115 (33.2%) patients. Delirium was present in 83 out of 231 (36%) patients with aphasia and 32 out of 115 (27.8%) patients without aphasia (*p* = 0.132). For patients without aphasia, sensitivity and specificity at the established cut-off value of ≥ 4 points were 100% and 78%, respectively. For patients with aphasia, the test demonstrated inferior performance, with a sensitivity and specificity of 98% and 55%, respectively. It was necessary to increase the cut-off value to ≥ 5 points. Through this, sensitivity was 90% (95% CI, 81.9–95.8%) and specificity was 75% (95% CI, 67.2–81.8%). The degree of agreement to the DSM-V criteria was “substantial” (Cohen's κ = 0.61).

**Conclusion:** For the purpose of delirium screening in patients with aphasia, increasing the ICDSC cut-off value to ≥ 5 points enables effective screening. Further studies are necessary to characterize post-stroke delirium.

## Introduction

Delirium is characterized by an acute, fluctuating and reversible state of inattention, confusion or an altered level of consciousness. Essentially, delirium is “a decompensation of cerebral function in response to one or more pathophysiological stressors” (1). The recently updated National Institute for Health and Care Excellence (NICE) guidelines recognize that patients with delirium have a longer duration of hospital and intermediate care stay (2), increased incidence of dementia, higher rates of hospital-acquired complications (e.g., falls, pressure sores), are more likely to be admitted to long-term care after hospital and have a higher mortality (3).

To prevent these complications, delirium has to be detected early through validated screening tools to enable consequent treatment. To this end, two delirium screening tools are commonly used: the Confusion Assessment Method for the Intensive Care Unit (CAM-ICU) and the Intensive Care Delirium Screening Checklist (ICDSC) (4). The CAM-ICU encompasses a letters attention test (“Whenever you hear the letter ‘A,’ squeeze my hand.”), Yes-no questions (“Will a stone float on water? Are there fish in the sea?”), and commands (“Hold up this many fingers.”). The ICDSC instead uses open questions (Item 3, “Disorientation”) and passive assessment of speech (Item 6, “Inappropriate speech or mood”). Additional non-verbal features have made the ICDSC more accurate than the CAM-ICU in the assessment of delirium in a small cohort of patients with intracranial hemorrhage (5).

There continues to be a need for validated and feasible delirium screening tools for neurologically critically ill patients (6). Specifically, patients with acute stroke are commonly affected by both delirium (10–30%) (7), and aphasia (21–38%) (8). This provides a challenge, as all available delirium screening tools require some degree of language function. Hence, it is unclear whether delirium screening tools like the ICDSC are valid in aphasic patients.

## Materials and Methods

### Study Design

Single-center prospective cohort study.

### Participants

The study was carried out in all adult patients consecutively admitted to the Stroke Unit (SU) at University Hospital Tuebingen between July 2017 and December 2018. Exclusion criteria were: (i) a duration of stay in the Stroke Unit of <24 h; (ii) a Richmond Agitation-Sedation Scale (RASS) level of −5 or −4 for the majority (>50%) of the stay; (iii) patients on mechanical ventilation or in shock; (iv) diagnosis of delirium or exposure to benzodiazepines on admission; (v) an incomplete record of the National Institutes of Health Stroke Scale (NIHSS), RASS and ICDSC during the stay. Of the 1,737 screened patients, 346 patients were eligible for further analysis.

### Test Methods

The index test was the Intensive Care Delirium Screening Checklist (ICDSC), administered on admission and every 8 h for the whole duration of the stay in the Stroke Unit. The ICDSC encompasses eight items, including: alteration of consciousness level, inattention, disorientation, hallucination or psychosis, psychomotor agitation or retardation, inappropriate speech or mood, sleep/wake cycle disturbance, and symptom fluctuation. Absence of symptoms is scored with 0 and presence of symptoms is scored with 1 point in each of the items. A cut-off score of 4 points is considered to be indicative of delirium, while a score of >4 points does not reflect the severity of delirium (9), but may be an independent risk factor for longer hospital stay (2). The ICDSC was administered by neurocritical care nurses trained in its use. The interrater agreement of the ICDSC has been demonstrated elsewhere (10).

Delirium assessment based on the Diagnostic and Statistical Manual of the American Psychiatric Association (DSM-V) diagnostic criteria was the reference standard. We additionally gave consideration to information obtained from third-party medical history, previous observations on the patient's behavior and records on pre-admission cognitive status, if available. Assessment was performed by an independent consultant neurologist, who was blinded for index test results of the ICDSC.

The Richmond Agitation-Sedation Scale (RASS) and the National Institutes of Health Stroke Scale (NIHSS) were assessed upon admission and every 6 h for the whole duration of SU stay. Assessment of language function was carried out by a consultant neurologist as part of the NIHSS.

### Statistical Analysis

To compare measures of diagnostic accuracy, cross tabulation for two categorical variables was carried out. Sensitivity (TPR), specificity (TNR), positive predictive value (PPV), and negative predictive value (NPV) were calculated.

Descriptive statistical analysis was carried out to determine differences in the baseline characteristics between aphasic and non-aphasic patients. Student's *t*-test and Pearson's chi-squared test were used where appropriate. The significance level was set at *p* < 0.05.

Cohen's Kappa (κ) was calculated to measure the degree of agreement between the gold-standard DSM-V diagnostic criteria and diagnosis of delirium as per a given ICDSC cut-off value. The strength of agreement, denoted by ranges of Kappa statistics, was labeled as proposed by Landis and Koch (11). Statistical analyses were performed with the SPSS 22.0 (IBM, Amonk, NY, USA).

### Ethics Approval and Consent

The study was approved by the ethics committee of the University Hospital Tuebingen (752/2018BO2). Informed consent was waived for this study.

## Results

Three hundred and forty six patients were included in the analysis. On admission or during hospital stay, aphasia was present in 231 (66.8%) and absent in 115 (33.2%) patients on admission. There was no significant difference in age and gender distribution between groups. The demographic and medical characteristics of the study population are given in Table 1.

**Table 1:** Baseline demographic and clinical characteristics of the study population.

	**Patients without aphasia (*n* = 115)**	**Patients with aphasia (*n* = 231)**	***p*-value**
Age (years)	74 (20–94; SD 15.3)	76 (26–100; SD 12.9)	0.203^a^
Gender	53% female, 47% male	50% female, 50% male	0.602^b^
Length of stay (days)	1.6 (1–5; SD 0.8)	4.6 (1–27; SD 3.9)	**<0.0001**^a^
RASS score	−0.1 (−5–4; SD 1.1)	−0.3 (−5–4; SD 1.9)	0.297^a^
NIHSS score	3.6 (0–21; SD 4.4)	12.3 (1–34; SD 7.0)	**<0.0001**^a^
ICDSC score	2.9 (0–8; SD 2.5)	4.4 (1–8; SD 2.1)	**<0.0001**^a^
Diagnosis of TIA	27 (23%)	8 (3.5%)	**<0.00001**^b^
Diagnosis of AIS	77 (67%)	174 (75%)	0.100^b^
Diagnosis of ICH	7 (6%)	41 (18%)	**0.003**^b^

a *Student's t-test*.

b *Pearson's chi-squared test*.

*Ranges and standard deviation are reported in brackets. RASS, Richmond Agitation-Sedation Scale; NIHSS, National Institutes of Health Stroke Scale; TIA, transient ischemic attack; AIS, acute ischemic stroke; ICH, intracranial hemorrhage*.

*Bold p-values indicate statistical significance at a 95% confidence interval*.

**Figure 1:**
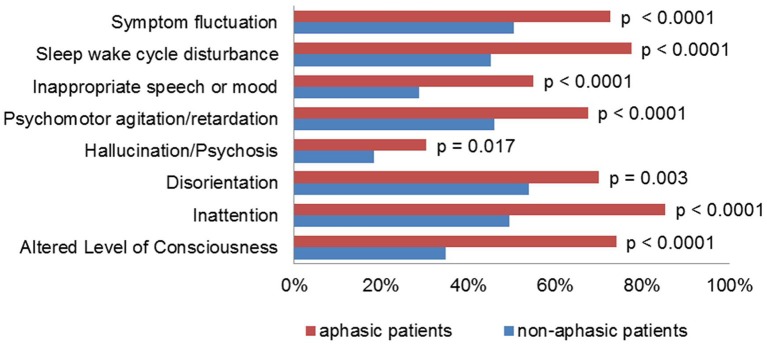
Frequency of ICDSC items in aphasic and non-aphasic patients.

Patients with aphasia scored higher than patients without aphasia on the NIHSS (12.3 vs. 3.6 mean score) and spent a significantly longer time in the intensive care unit (4.6 vs. 1.6 days mean length of stay, *p* < 0.0001). The average ICDSC score was significantly higher in patients with aphasia, and every item on the ICDSC contributed significantly more often to the score (Figure 1). Of these, the items “altered level of consciousness” and “inappropriate speech or mood” were much more prevalent in patients with aphasia, as compared to patients without aphasia (74% vs. 35% and 55% vs. 29%, respectively).

The target condition, delirium, according to the reference standard DSM-V diagnostic criteria, was present in 83 out of 231 (36%) patients with aphasia and 32 out of 115 (27.8%) patients without aphasia (*p* = 0.132). Cross tabulation of the index test results in patients with and without aphasia is given in Table 2.

**Table 2:** ICDSC test results in aphasic and non-aphasic patients at given cut-off values.

			**DSM-V**		**PPV**	**NPV**	**TPR**	**TNR**	**κ**
			**+**	**–**					
Patients with aphasia (*n* = 231)	ICDSC ≥ 4	+	81	66	55% [50.6–59.6]	98% [91.2–99.4]	98% [91.6–99.7]	55% [47.02–63.6]	0.45
		–	2	82					
	**ICDSC** **≥** **5**	+	75	37	**67% [60.3–72.9]**	**93% [87.7–96.4]**	**90% [81.9–95.8]**	**75% [67.2–81.8]**	**0.61**
		–	8	111					
	ICDSC ≥ 6	+	62	18	78% [68.7–84.4]	86% [81.0–90.0]	75% [63.9–83.6]	88% [81.5–93.6]	0.63
		–	21	130					
Patients without aphasia (*n* = 115)	ICDSC ≥ 3	+	32	29	52% [45.1–59.7]	100%	100% [89.1–100]	65% [53.8–75.2]	0.51
		–	0	54					
	**ICDSC** **≥** **4**	+	32	18	**64% [54.2–72.8]**	**100%**	**100%** [89.1–100]	**78% [67.9–86.6]**	**0.67**
		–	0	65					
	ICDSC ≥ 5	+	26	7	79% [64.2–88.5]	93% [86.0–96.3]	81% [63.6–92.8]	92% [83.4–96.5]	0.72
		–	6	76					
	ICDSC ≥ 6	+	17	3	85% [64.0–94.8]	84% [78.6–88.6]	53% [34.7–70.9]	96% [89.8–99.3]	0.56
		–	15	80					

*95% confidence intervals are reported in square brackets. PPV, positive predictive value; NPV, negative predictive value; TPR, true positive rate or sensitivity; TNR, true negative rate or specificity; κ, Cohen's Kappa*.

*Bold values indicate the ICDSC cut-off values proposed by the authors*.

For patients without aphasia, sensitivity and specificity of the ICDSC were 100% (95% CI, 89.1–100%) and 78% (95% CI, 67.2–81.8%), respectively. The degree of agreement to the DSM-V diagnostic criteria was “substantial” (Cohen's κ = 0.67). Increasing the cut-off value by 1 point resulted in a decrease of sensitivity from 100% to 81%.

For patients with aphasia, specificity of the ICDSC at a cut-off value of ≥ 4 points was 55% (95% CI, 47.0–63.6%). The degree of agreement to the DSM-V criteria was “moderate” (Cohen's κ = 0.45). However, at a cut-off value of ≥ 5 points on the ICDSC, specificity increased to 75% (95% CI, 67.2–81.8%), sensitivity was still at 90% (95% CI, 81.9–95.8%), and the degree of agreement to the DSM-V criteria was “substantial” (Cohen's κ = 0.61).

Further subgroup analysis on aphasia severity was performed. The ICDSC test performance was worse in patients with global aphasia (NIHSS-Item 9, “Best Language”) (12). At a cut-off value of ≥ 5 points, sensitivity was 71% and specificity was 91%. Eight aphasic patients with delirium were not detected. On review of these cases, three of these patients had suffered major ischemic stroke (NIHSS ≥ 20 points) in the left medial cerebral artery (MCA) territory. Results of this subgroup analysis are provided as Supplementary Material.

## Discussion

Language is disrupted by both post-stroke delirium and post-stroke aphasia. In delirium, a number of higher cognitive functions are affected, including working memory, attention and visuospatial processing. While the impact of delirium on language function has been recognized in all major classification systems (DSM-III, DSM-III-R, DSM-V, and ICD-10), the literature on language dysfunction in delirious patients is scarce (13, 14).

Delirium screening is challenging if speech comprehension or speech production are impaired. In a survey of healthcare providers working in acute stroke, the majority of respondents reported difficulties using delirium screening tools in aphasic patients (15). Furthermore, patients with aphasia have previously been excluded from a number of studies on post-stroke delirium, leading to exclusion bias (16).

In our study, we found that the established cut-off value of ≥4 points on the ICDSC was not suitable for the detection of delirium in patients with aphasia, since the specificity dropped to 55%. These patients had a higher average ICDSC score and every item on the ICDSC contributed more often to the score, especially for “altered level of consciousness” and “inappropriate speech or mood”. To reflect this, we propose to raise the cut-off value of the ICDSC to ≥5 points for patients with aphasia. In our cohort, this resulted in a sensitivity and specificity of 90% and 75%, respectively. This compares favorably to a previous meta-analysis on the ICDSC in delirium screening, in which the pooled sensitivity and specificity were 74% and 82%, respectively (4).

A number of limitations apply. The study setting was a Stroke Unit and our results may not be applicable in other settings. Notably, aphasia was much more prevalent in our patients than in previous cohorts (66.8% vs. 21–38%) (7). Sampling bias may have been introduced by excluding patients with an incomplete score record (NIHSS, RASS, and ICDSC), as this is more likely to be true for non-aphasic or non-delirious patients. Presence and severity of aphasia was based on the NIHSS (Item 9, “Best Language”), but no information was obtained on the type of aphasia. The reference standard was assessed by a neurologist, not a psychiatrist. No information was available on delirium subtype (hyperactive, hypoactive, mixed) or severity of delirium. Care should be taken when assessing patients with major ischemic stroke (NIHSS ≥ 20 points) in the left MCA territory or global aphasia: Our results suggest that underdetection of delirium is likely in these patients.

## Conclusion

Delirium screening of aphasic patients is a diagnostic dilemma which is encountered often in neurocritical care. To our knowledge, this is the first study to examine the performance of this delirium screening tool in aphasic patients. For effective delirium screening in aphasic patients, the ICDSC cut-off value has to be increased from ≥4 points to ≥5 points.

Our results may improve delirium screening sensitivity, assisting healthcare professionals in recognizing delirium in patients with aphasia. This also enables further studies of this cohort and may aid in characterizing post-stroke delirium.

## Data Availability Statement

The datasets generated for this study are available on request to the corresponding author.

## Ethics Statement

The studies involving human participants were reviewed and approved by University Hospital Tuebingen (752/2018BO2). The patients/participants provided their written informed consent to participate in this study.

## Author Contributions

CB and AM created concept and design of the study, jointly acquired, and analyzed the data and wrote the manuscript. JZ and VS acquired and analyzed the data and critically reviewed the manuscript. M-IS, HL, YW, SP, and UZ helped with the study design and critically reviewed the manuscript.

### Conflict of Interest

SP received speaker's honoraria and consulting honoraria from Bayer, Boehringer-Ingelheim, Bristol-Myers Squibb/Pfizer, Daiichi Sankyo, and Werfen, reimbursement for congress traveling and accommodation from Bayer and Boehringer-Ingelheim, and research support from Bristol-Myers Squibb/Pfizer (significant), Boehringer-Ingelheim, Daiichi Sankyo (significant), and Helena Laboratories (all other contributions: modest). All competing interest are outside of the present work. UZ has received grants from European Research Council, German Research Foundation, German Ministry of Education and Research, Biogen Idec GmbH, Servier, and Janssen Pharmaceuticals NV, all not related to this work; and consulting honoraria from Biogen Idec GmbH, Bayer Vital GmbH, Bristol Myers Squibb GmbH, Pfizer, CorTec GmbH, Medtronic GmbH, all not related to this work. HL received grants from the German Research Foundation (DFG), the German Federal Ministry of Education and Research (BMBF), Bial, and speaker's or consulting honoraria or travel support from Bial, BioMarin, Desitin, Eisai, and UCB, all not related to the current work. The remaining authors declare that the research was conducted in the absence of any commercial or financial relationships that could be construed as a potential conflict of interest.
