# Supplementary material for: Delirium Screening in Aphasic Patients With the Intensive Care Delirium Screening Checklist (ICDSC): A Prospective Cohort Study
Source: Front Neurol. 2019 Nov 12;10:1198. doi: 10.3389/fneur.2019.01198 (PMC6861445; doi:10.3389/fneur.2019.01198)
Supplement: Supplementary file 1 [file Table_1.DOCX]

**Supplementary Material**

| **NIHSS-Aphasia 0-3** | | **ICDSC ≥ 3** | |  |  | PPV | 48% |
| --- | --- | --- | --- | --- | --- | --- | --- |
|  |  | **+** | **-** |  |  | NPV | 99% |
| **DSM** | **+** | 114 | 1 | 115 |  | TPR | 99% |
|  | **-** | 125 | 106 | 231 |  | TNR | 46% |
|  |  | 239 | 107 | 346 |  | Cohen's κ | 0,35 |
|  |  |  |  |  |  |  |  |
| **NIHSS-Aphasia 0-3** | | **ICDSC ≥ 4** | |  |  | PPV | 57% |
|  |  | **+** | **-** |  |  | NPV | 99% |
| **DSM** | **+** | 113 | 2 | 115 |  | TPR | 98% |
|  | **-** | 84 | 147 | 231 |  | TNR | 64% |
|  |  | 197 | 149 | 346 |  | Cohen's κ | 0,52 |
|  |  |  |  |  |  |  |  |
| **NIHSS-Aphasia 0-3** | | **ICDSC ≥ 5** | |  |  | PPV | 72% |
|  |  | **+** | **-** |  |  | NPV | 93% |
| **DSM** | **+** | 111 | 14 | 115 |  | TPR | 97% |
|  | **-** | 44 | 187 | 231 |  | TNR | 81% |
|  |  | 155 | 201 | 346 |  | Cohen's κ | 0,70 |
|  |  |  |  |  |  |  |  |
| **NIHSS-Aphasia 0-3** | | **ICDSC ≥ 6** | |  |  | PPV | 79% |
|  |  | **+** | **-** |  |  | NPV | 85% |
| **DSM** | **+** | 79 | 36 | 115 |  | TPR | 69% |
|  | **-** | 21 | 210 | 231 |  | TNR | 91% |
|  |  | 100 | 246 | 346 |  | Cohen's κ | 0,62 |
|  |  |  |  |  |  |  |  |
| **NIHSS-Aphasia 1-3** | | **ICDSC ≥ 4** | |  |  | PPV | 55% |
|  |  | **+** | **-** |  |  | NPV | 98% |
| **DSM** | **+** | 81 | 2 | 83 |  | TPR | 98% |
|  | **-** | 66 | 82 | 148 |  | TNR | 55% |
|  |  | 147 | 84 | 231 |  | Cohen's κ | 0,45 |
|  |  |  |  |  |  |  |  |
| **NIHSS-Aphasia 1-3** | | **ICDSC ≥ 5** | |  |  | PPV | 67% |
|  |  | **+** | **-** |  |  | NPV | 93% |
| **DSM** | **+** | 75 | 8 | 83 |  | TPR | 90% |
|  | **-** | 37 | 111 | 148 |  | TNR | 75% |
|  |  | 112 | 119 | 231 |  | Cohen's κ | 0,61 |
|  |  |  |  |  |  |  |  |
| **NIHSS-Aphasia 1-3** | | **ICDSC ≥ 6** | |  |  | PPV | 78% |
|  |  | **+** | **-** |  |  | NPV | 86% |
| **DSM** | **+** | 62 | 21 | 83 |  | TPR | 75% |
|  | **-** | 18 | 130 | 148 |  | TNR | 88% |
|  |  | 80 | 151 | 231 |  | Cohen's κ | 0,63 |
|  |  |  |  |  |  |  |  |
| **NIHSS-Aphasia 0** | | **ICDSC ≥ 3** | |  |  | PPV | 52% |
|  |  | **+** | **-** |  |  | NPV | 100% |
| **DSM** | **+** | 32 | 0 | 32 |  | TPR | 100% |
|  | **-** | 29 | 54 | 83 |  | TNR | 65% |
|  |  | 61 | 54 | 115 |  | Cohen's κ | 0,51 |
|  |  |  |  |  |  |  |  |
| **NIHSS-Aphasia 0** | | **ICDSC ≥ 4** | |  |  | PPV | 64% |
|  |  | **+** | **-** |  |  | NPV | 100% |
| **DSM** | **+** | 32 | 0 | 32 |  | TPR | 100% |
|  | **-** | 18 | 65 | 83 |  | TNR | 78% |
|  |  | 50 | 65 | 115 |  | Cohen's κ | 0,67 |
|  |  |  |  |  |  |  |  |
| **NIHSS-Aphasia 0** | | **ICDSC ≥ 5** | |  |  | PPV | 79% |
|  |  | **+** | **-** |  |  | NPV | 93% |
| **DSM** | **+** | 26 | 6 | 32 |  | TPR | 81% |
|  | **-** | 7 | 76 | 83 |  | TNR | 92% |
|  |  | 33 | 82 | 115 |  | Cohen's κ | 0,72 |
|  |  |  |  |  |  |  |  |
| **NIHSS-Aphasia 0** | | **ICDSC ≥ 6** | |  |  | PPV | 85% |
|  |  | **+** | **-** |  |  | NPV | 84% |
| **DSM** | **+** | 17 | 15 | 32 |  | TPR | 53% |
|  | **-** | 3 | 80 | 83 |  | TNR | 96% |
|  |  | 20 | 95 | 115 |  | Cohen's κ | 0,56 |
|  |  |  |  |  |  |  |  |
| **NIHSS-Aphasia 1** | | **ICDSC ≥ 4** | |  |  | PPV | 56% |
|  |  | **+** | **-** |  |  | NPV | 97% |
| **DSM** | **+** | 20 | 1 | 21 |  | TPR | 95% |
|  | **-** | 16 | 31 | 47 |  | TNR | 66% |
|  |  | 36 | 32 | 69 |  | Cohen's κ | 0,50 |
|  |  |  |  |  |  |  |  |
| **NIHSS-Aphasia 1** | | **ICDSC ≥ 5** | |  |  | PPV | 68% |
|  |  | **+** | **-** |  |  | NPV | 95% |
| **DSM** | **+** | 19 | 2 | 21 |  | TPR | 90% |
|  | **-** | 9 | 38 | 47 |  | TNR | 81% |
|  |  | 28 | 40 | 69 |  | Cohen's κ | 0,64 |
|  |  |  |  |  |  |  |  |
| **NIHSS-Aphasia 1** | | **ICDSC ≥ 6** | |  |  | PPV | 70% |
|  |  | **+** | **-** |  |  | NPV | 85% |
| **DSM** | **+** | 14 | 7 | 21 |  | TPR | 67% |
|  | **-** | 6 | 41 | 47 |  | TNR | 87% |
|  |  | 20 | 48 | 69 |  | Cohen's κ | 0,54 |
|  |  |  |  |  |  |  |  |
| **NIHSS-Aphasia 2** | | **ICDSC ≥ 4** | |  |  | PPV | 60% |
|  |  | **+** | **-** |  |  | NPV | 100% |
| **DSM** | **+** | 21 | 0 | 21 |  | TPR | 100% |
|  | **-** | 14 | 20 | 34 |  | TNR | 59% |
|  |  | 35 | 20 | 55 |  | Cohen's κ | 0,52 |
|  |  |  |  |  |  |  |  |
| **NIHSS-Aphasia 2** | | **ICDSC ≥ 5** | |  |  | PPV | 70% |
|  |  | **+** | **-** |  |  | NPV | 100% |
| **DSM** | **+** | 21 | 0 | 21 |  | TPR | 100% |
|  | **-** | 9 | 25 | 34 |  | TNR | 74% |
|  |  | 30 | 25 | 55 |  | Cohen's κ | 0,68 |
|  |  |  |  |  |  |  |  |
| **NIHSS-Aphasia 2** | | **ICDSC ≥ 6** | |  |  | PPV | 76% |
|  |  | **+** | **-** |  |  | NPV | 93% |
| **DSM** | **+** | 19 | 2 | 21 |  | TPR | 90% |
|  | **-** | 6 | 28 | 34 |  | TNR | 82% |
|  |  | 25 | 30 | 55 |  | Cohen's κ | 0,70 |
|  |  |  |  |  |  |  |  |
| **NIHSS-Aphasia 3** | | **ICDSC ≥ 4** | |  |  | PPV | 53% |
|  |  | **+** | **-** |  |  | NPV | 97% |
| **DSM** | **+** | 40 | 1 | 41 |  | TPR | 98% |
|  | **-** | 36 | 31 | 67 |  | TNR | 46% |
|  |  | 76 | 32 | 108 |  | Cohen's κ | 0,38 |
|  |  |  |  |  |  |  |  |
| **NIHSS-Aphasia 3** | | **ICDSC ≥ 5** | |  |  | PPV | 65% |
|  |  | **+** | **-** |  |  | NPV | 89% |
| **DSM** | **+** | 35 | 6 | 41 |  | TPR | 85% |
|  | **-** | 19 | 48 | 67 |  | TNR | 72% |
|  |  | 54 | 54 | 108 |  | Cohen's κ | 0,54 |
|  |  |  |  |  |  |  |  |
| **NIHSS-Aphasia 3** | | **ICDSC ≥ 6** | |  |  | PPV | 85% |
|  |  | **+** | **-** |  |  | NPV | 84% |
| **DSM** | **+** | 29 | 12 | 41 |  | TPR | 71% |
|  | **-** | 5 | 61 | 67 |  | TNR | 91% |
|  |  | 34 | 73 | 108 |  | Cohen's κ | 0,64 |

**Supplementary Material 1:** ICDSC test results at given cut-off values. Subgroup analysis of aphasia severity as reported on the NIHSS. Abbreviations: PPV – positive predictive value, NPV – negative predictive value, TPR – true positive rate or sensitivity, TNR – true negative rate or specificity, κ – Cohen’s Kappa.
